# Chronic iron exposure and c-Myc/H-ras-mediated transformation in fallopian tube cells alter the expression of EVI1, amplified at 3q26.2 in ovarian cancer

**DOI:** 10.1038/s41389-019-0154-y

**Published:** 2019-08-21

**Authors:** Stephanie Rockfield, Younghoon Kee, Meera Nanjundan

**Affiliations:** Department of Cell Biology, Microbiology, and Molecular Biology, University of South Florida, Tampa, FL USA

**Keywords:** Ovarian cancer, Cell growth

## Abstract

Mechanisms underlying the pathogenesis of high-grade serous epithelial ovarian cancers (HGSOC) are not yet well defined although key precursor cells have been identified (including fimbriated fallopian tube epithelium, FTSECs). Since iron is elevated in endometriotic cysts and the pelvic cavity, it is suggested that this source of redox-active iron may contribute to ovarian cancer pathogenesis. Specifically, sources of nontransferrin-bound iron (NTBI) within the pelvic cavity could arise from ovulation, retrograde menstruation, follicular fluid, or iron overload conditions (i.e., hemochromatosis). Herein, we investigated the cellular response of p53-inactivated and telomerase-expressing (immortalized) FTSECs (Pax8^+^/FoxJ1^−^) to NTBI (presented as ferric ammonium citrate (FAC), supplemented in media for >2 months) in order to assess its ability to promote the transition to a tumor-like phenotype; this cellular response was compared with immortalized FTSECs transformed with H-Ras^V12A^ and c-Myc^T58A^. Both approaches resulted in increased cell numbers and expression of the oncogenic transcriptional regulator, ecotropic virus integration site 1 (EVI1, a gene most frequently amplified at 3q26.2 in HGSOC, represented by multiple variants), along with other oncogenic gene products. In contrast to the transformed cells, FAC-exposed FTSECs elicited elevated migratory capacity (and epithelial–mesenchymal transition mRNA profile) along with increased expression of DNA damage response proteins (i.e., FANCD2) and hTERT mRNA relative to controls. Interestingly, in FAC-exposed FTSECs, EVI1 siRNA attenuated hTERT mRNA expression, whereas siRNAs targeting β-catenin and BMI1 (both elevated with chronic iron exposure) reduced Myc and Cyclin D1 proteins. Collectively, our novel findings provide strong foundational evidence for potential iron-induced initiation events, including EVI1 alterations, in the pathogenesis of HGSOC, warranting further in depth investigations. Thus, these findings will substantially advance our understanding of the contribution of iron enriched within the pelvic cavity, which may identify patients at risk of developing this deadly disease.

## Introduction

Ovarian cancer (OVCA) is the 5th deadliest cancer, and the most lethal cancer in women in the United States^1^. The etiology of this disease remains unclear, which has made it difficult to improve early detection and therapeutic strategies for these patients^2–4^. Recent research supports the hypothesis that high-grade serous epithelial ovarian cancer (HGSOC, the most commonly diagnosed subtype^3^) may arise from fallopian tube epithelium (FTE^5^) or from ovarian surface epithelium (OSE)^6^. Well-defined characteristics of HGSOC include inactivated p53, mutant BRCA1/2, homozygous deletion of PTEN, hyperactive Ras-MAPK signaling, elevated expression of Pax8 (paired box protein 8, a transcription factor), and elevated human telomerase reverse transcriptase (hTERT)^7^. In addition, TCGA-defined genomic amplifications include those at 3q26 (harboring ecotropic viral integration site 1 (EVI1) amongst other genes including the RNA component of telomerase (TERC)) and 8q24 (harboring the proto-oncogene c-Myc)^5,7–13^. However, the early events that mediate the transition from precursors such as fallopian tube secretory epithelial cells (FTSECs) to HGSOC have yet to be determined.

Catalytic iron, which mediates the generation of reactive oxygen species (ROS) through its involvement in Fenton reactions leading to lipid peroxidation and DNA damage^14^, can contribute to the pathogenesis of specific cancer types such as colorectal cancers^15^. Specifically in epithelial OVCAs, it is well defined that rare subtypes including endometrioid and clear cell may develop as a result of prolonged exposure to elevated catalytic iron present within endometriotic cysts (proposed precursor lesion)^16,17^. Other sources of microenvironmental catalytic iron within the pelvic cavity (i.e., accumulating within the Douglas Pouch) could arise due to ovulation, retrograde menstruation, and follicular rupture; elevated systemic iron may arise as a result of an iron overload condition such as hemochromatosis^18–21^. Iron overload is characterized by elevated nontransferrin-bound iron (NTBI)^22^ and ferric ammonium citrate (FAC) is an appropriate source to investigate its effect^23^. To our knowledge, NTBI has not yet been previously investigated as a factor involved in OVCA pathogenesis although a positive correlation between hemochromatosis and OVCA risk has been reported^24^. Although recently published data using immortalized FTSECs demonstrate that transferrin-bound iron can increase intracellular ROS and DNA damage^25^ and a NTBI source increases cellular proliferation in primary fimbrial cells (associated with increased c-Myc, MAPK, and AKT activation)^26^, the contribution of physiological NTBI to HGSOC disease pathogenesis remains uninvestigated.

Herein, we chronically maintained two proposed precursors to HGSOC (immortalized OSE cells (T80) or immortalized FTSECs) in media supplemented with FAC between 2 and 4 months. We compared their outcome with a FTSEC cell line that we transformed by overexpressing the constitutively active forms of H-Ras and c-Myc. While T80 cells showed reduced cell numbers in response to chronic NTBI treatment, we observed increased cell numbers, migratory capacity, and increased DNA damage/repair proteins (i.e., FANCD2) in FAC-treated relative to untreated FTSECs (maintained concurrently in culture). This observation suggests that the iron effect is specific to FTSECs. Although the transformed FTSECs similarly showed increased clonogenic potential and cell numbers, they displayed reduced cellular migration capacity. Assessment of the expression of oncogenes frequently associated with HGSOC (including EVI1 variants, Myc, BMI1, and Cyclin D1) identified that these were markedly elevated in FTSECs with either chronic iron treatment or cellular transformation. We also noted differences between their expression profiles of hTERT mRNA and β-catenin protein, suggesting that the observed functional outcomes may be mediated via different mechanisms. EVI1 knockdown (using siRNA targeting multiple splice variants) attenuated the increased hTERT mRNA expression upon chronic iron treatment, whereas EVI1 itself was regulated independently of β-catenin, BMI1, or autophagic pathways. The observed changes in EVI1 and Myc were not reversed when chronic FAC-treated cells were maintained in FAC-deficient media suggesting the acquisition of permanently acquired alterations. Together, our results suggest that iron may enable the transition of fallopian tube precursor lesions via altering the expression patterns for some of the key oncogenic factors identified in OVCA. Thus, the presence of iron within the pelvic cavity may be a potential risk factor in disease development and could be targeted as a strategy to reduce tumor burden.

## Results

### Generation of chronic iron-exposed and transformed FTSECs

Although evidence exists that links elevated free iron (NTBI) to OVCA pathogenesis^16,17,21,24,27^, the mechanisms underlying its contribution have yet to be defined experimentally. Since it is theorized that the cell of origin for HGSOC may derive from either the OSE or the FTE^5,6^, we thus utilized immortalized human ovarian surface epithelial cells (T80, which stably express SV40 Large T Antigen (which inactivates p53 and Rb) and hTERT) as well as immortalized FT194 (which were also generated via stable expression of SV40 LTAg and hTERT^28^) to investigate the long-term effects of NTBI exposure. Indeed, precursor lesions of the fallopian tube contain p53 signatures (enriched in inactivated p53^29^) and 95% of HGSOC cases are characterized by p53 inactivating mutations^7^ and thus, the effect of long-term iron exposure under these conditions was investigated. However, maintaining T80 cells for nearly 200 days in the presence of 250 μM FAC (as a source of NTBI, using a dose previously identified to not alter short-term T80 cell viability^30^) resulted in reduced cell numbers (*p* = 0.2447, Supplementary Fig. 1).

**Fig. 1:**
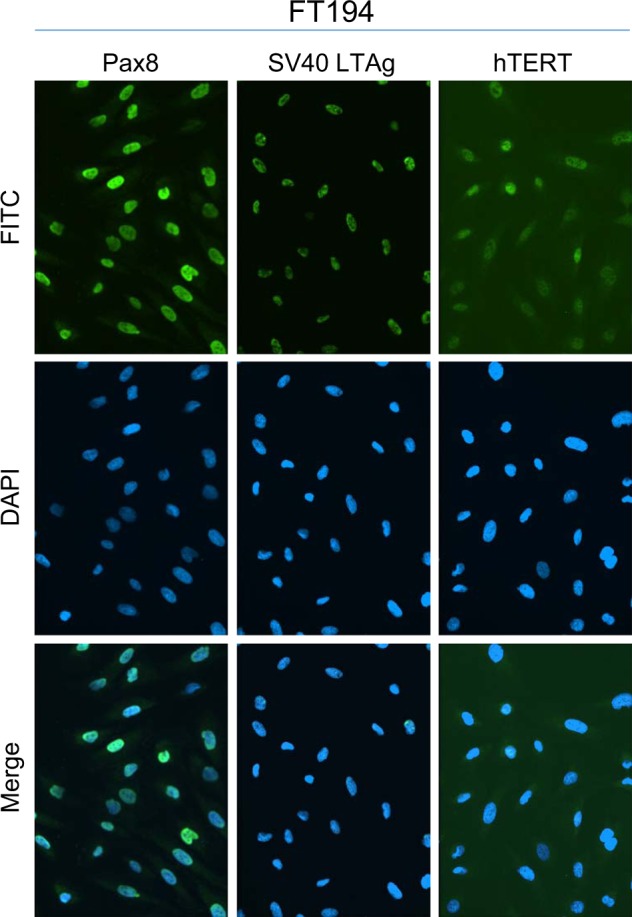
Characterization of parental FTSECs. Representative immunofluorescence images for Pax8, SV40 LTAg, and hTERT in FT194 cells (*p* = 15) captured at ×20 magnification

**Fig. 2:**
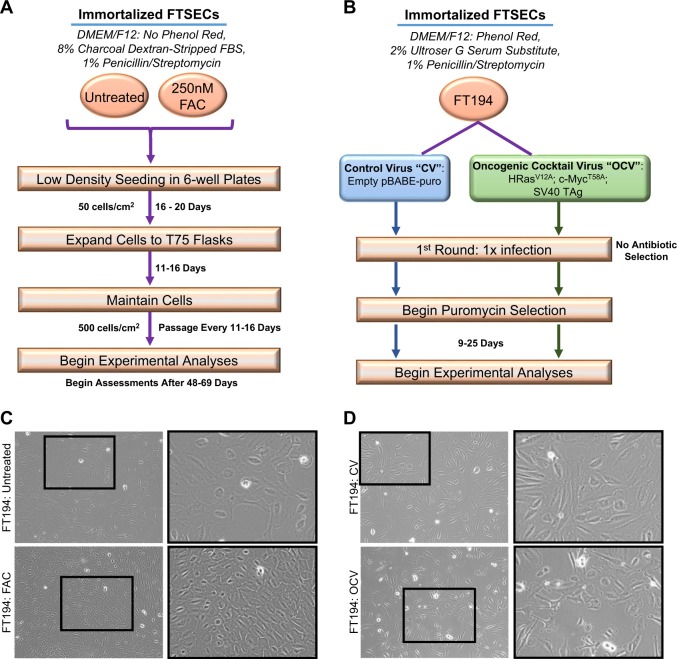
Generation of chronic iron-exposed and transformed FTSECs. **a** Schematic summarizing the maintenance of FT194 cells in FAC. **b** Schematic depicting the method for transforming FT194 cells via retroviral infection of H-Ras^V12A^, c-Myc^T58A^, and SV40 LTAg. **c** Representative light microscope images of FT194-untreated and 250 nM FAC-treated cells (*p* = 19). Images were captured at ×100 magnification and a subpanel of the image was enlarged in PowerPoint. **d** Representative light microscope images of FT194 control virus (CV) and oncogenic cocktail virus (OCV) cells (*p* = RV + 3). Images were captured at ×100 magnification and a subpanel of the image was enlarged in PowerPoint

Since chronic iron treatment in immortalized human OSE did not increase cell numbers, we next investigated its effect on immortalized FTSECs. We first confirmed that the phenotype of our FTSECs were positive for the Müllerian marker, Pax8, and negative for the ciliated cell marker, FoxJ1^8,28,31,32^. Indeed, we confirmed that FT194 cell lines were Pax8^+^ (100%) and FoxJ1^−^ in addition to expressing SV40 LTAg^+^ (~99%) and hTERT^+^ (Fig. 1; results for FoxJ1 were negative (as expected since ciliated FoxJ1^+^ FTSECs cannot be maintained in two-dimensional culture systems^28^) (results not shown)). To next investigate whether iron may contribute in the transition of fallopian tube precursors to HGSOC, we chronically maintained FT194 cells in culture media supplemented with FAC (see “Materials and methods” section for additional details); Fig. 2a summarizes details for cell handling of FTSECs. Briefly, we began by seeding cells at low density, then supplemented the media with a range of FAC doses based on physiological levels of NTBI and prior supporting literature^17,30,33–35^. Within ~2 weeks, we observed a reduction in cell size as well as increased cell numbers of FT194 cells treated with 250 nM FAC (relative to untreated cells maintained concurrently in culture). In addition, we generated transformed FTSECs through retroviral transduction of an “oncogenic cocktail virus (OCV)” consisting of c-Myc^T58A^, H-Ras^V12A^, and SV40 LTAg cDNAs, using previously published methods^36,37^. A schematic of the transformation process is depicted in Fig. 2b. Representative light microscope images for FT194 untreated and FAC cells (Fig. 2c) as well as FT194 control virus-infected (CV) and OCV-infected cell lines (Fig. 2d) displays morphological changes after 60 days of chronic 250 nM FAC treatment and after integration of the transduced oncogenes, respectively. Specifically, the untreated and CV-infected FT194 cells were egg-shaped and more extensively flattened compared with the FAC and OCV-infected cells.

**Fig. 3:**
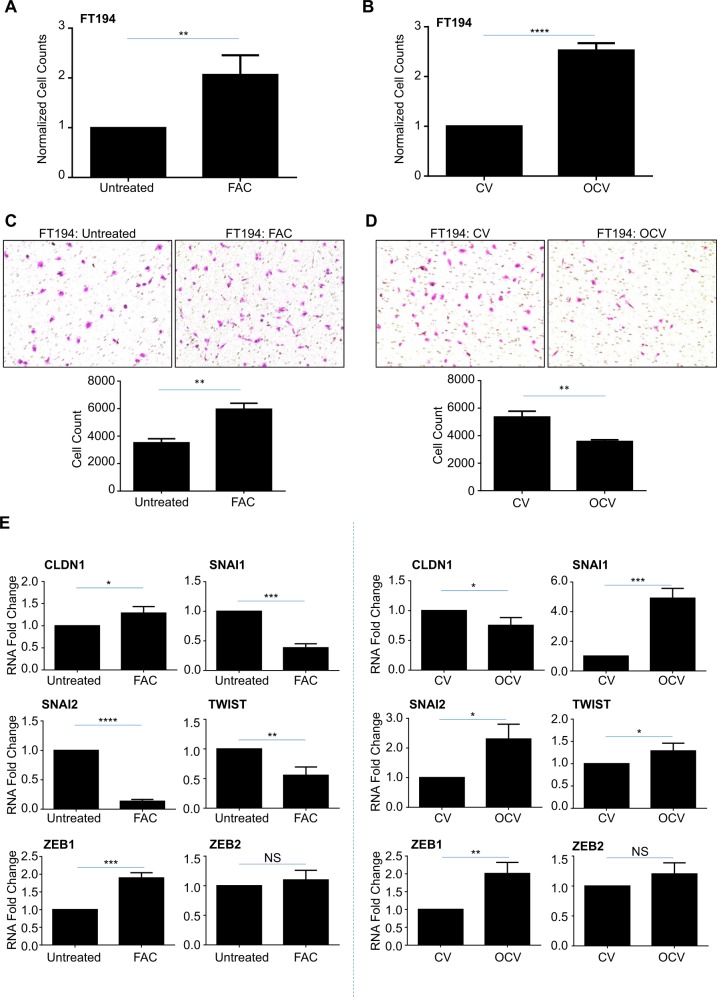

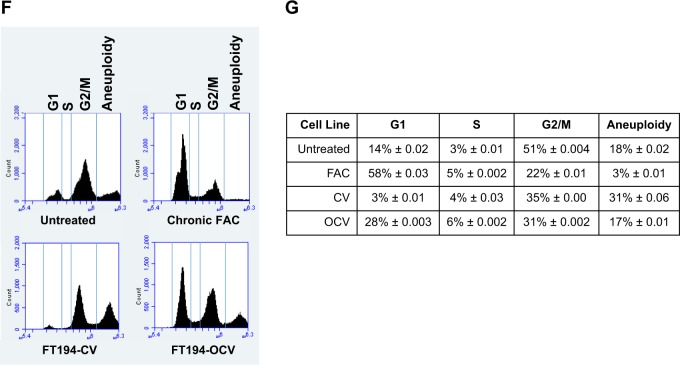
Chronic iron exposure and transformed FTSECs elicit altered migratory and proliferative capacities. **a** Untreated and 250 nM FAC-treated FT194 cells were seeded at 500 cells/cm^2^ and maintained over a period of 35–134 days (*p* = 17–39). Representative cell counts from untreated and FAC-treated cells on days 48, 67, 90, and 138 are presented. **b** FT194-CV and FT194-OCV cells were seeded at 500 cells/cm^2^ (*p* = RV + 7) and maintained in culture for 10 days prior to counting; the graph represents three independent replicates. Light microscope images of migrated untreated and FAC-treated (**c**; *p* = 30, day 104) as well as CV and OCV (**d**; *p* = RV + 10) FT194 cells. Images were captured at ×100 magnification, as detailed in the “Materials and methods” section. For quantification, migrated cells were counted from each independent replicate. **e** RNA was isolated from untreated and FAC-treated FT194 cells (*p* = 20, day 69) to assess mRNA expression of the indicated EMT markers via real-time PCR. Data represent three independent experiments. **f, g** Cell cycle analyses were completed for untreated and chronic FAC-treated FT194 cells (*p* = 21–22, days 71–74) as well as FT194-CV and FT194-OCV cells (*p* = RV + 3). For all panels, the results shown are representative of three independent experiments

We next assessed the clonogenic potential of these iron-treated and transformed FT194 cells by seeding cells at 500 cells/cm^2^ and monitoring colony growth. As shown in Supplementary Fig. 2a, untreated FTSECs grew in colonies although FAC-treated cells were more dispersed (particularly noted at low density), whereas FT194-OCV cells showed a marked increase in colony growth relative to CV (Supplementary Fig. 2b). We thus counted untreated, FAC-treated, FT194-CV, and FT194-OCV cells which were equivalently seeded at 500 cells/cm^2^ and identified a significant increase in cell numbers in FAC-treated relative to untreated FT194 cells (~twofold increase, *p* = 0.0015) and with FT194-OCV relative to FT194-CV (~2.5-fold increase, *p* < 0.0001; Fig. 3a, b), suggesting an increase in cell growth potential. We questioned whether these cell lines were also more migratory, and thus completed a migration assay using Boyden chambers. As shown in Fig. 3c, we observed a significant increase in cell migration with FAC-exposed relative to untreated cells (*p* = 0.0013); however, the migration capacity was significantly reduced in FT194-OCV relative to FT194-CV (*p* = 0.0022, Fig. 3d). Quantitative PCR analyses for markers of epithelial-to-mesenchymal transition (EMT) were next assessed in untreated to FAC-treated FT194 cells (Fig. 3e, left panels) as well as FT194-CV to FT194-OCV cells (Fig. 3e, right panels). In both comparisons, we identified a significant increase in the mRNA transcripts of ZEB1 (*p* = 0.0005, *p* = 0.0049, respectively) with no change in ZEB2 (*p* = 0.3592, *p* = 0.1302, respectively). Opposite trends in gene expression of Claudin-1 (CLDN1), SNAIL (SNAI1), SLUG (SNAI2), and TWIST were noted, which may correlate with the differing migratory capacity of the FAC-treated and FT194-OCV cells.

We next completed cell cycle analyses and identified that untreated FT194 cells contained a large proportion of aneuploid cells (>4N), whereas an aneuploid population was markedly reduced following chronic iron treatment (Fig. 3f). We compared this cell cycle profile with FT194-CV and FT194-OCV cells and noted similar changes in the number of aneuploid cells following OCV-induced transformation, although the number of aneuploid cells in the OCV population was more relative to FAC-exposed FT194 cells (likely due to the timewise maintenance of the cells in culture) (Fig. 3f, g). Although increased DNA content is a commonly accepted feature of cancer progression, aneuploidy has also been implicated in impairing cell proliferation and tumorigenesis^38^. Collectively, these results suggest that chronic iron treatment and oncogenic transformation of FTSECs increases cell numbers and may potentially select for cells lacking an aneuploid profile.

### Chronic iron-treated FTSECs display increased DNA damage, in contrast to transformed FTSECs

**Fig. 4:**
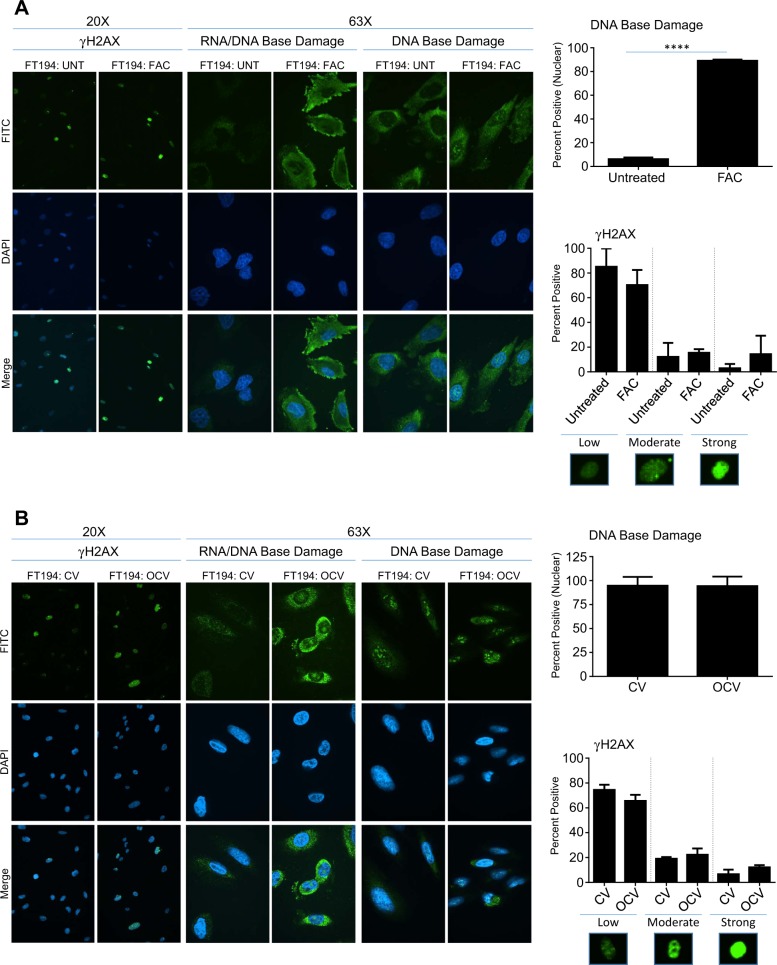
Chronic iron-exposed FTSECs display increased DNA damage. Immunofluorescence for γH2AX and 8-OHG (either without (middle panels) or with (right panels) RNase treatment) in untreated and FAC-treated (**a**; *p* = 26–28, days 90–97) as well as CV and OCV (**b**; *p* = RV + 7-RV + 8) FT194 cells. Representative images were captured at ×20 (γH2AX) or ×63 (oil objective, 8-OHG) magnification. Representative immunofluorescence images from FAC-treated (**a**) and FT194-OCV (**b**) representing each category (low, moderate, and strong) are also displayed

Iron is known to generate ROS, which subsequently promotes DNA damage^14,39^. We thus assessed, via an immunofluorescence approach, DNA damage in our long-term iron-exposed and transformed FT194 cells. As shown in Fig. 4a, there were mild increases in the levels of the DNA double stranded break marker (γH2AX) in FAC-treated cells (relative to untreated, likely due to the increased baseline level of DNA damage accumulation that can arise due to the process of immortalization (i.e., large T antigen^40^)). In contrast, DNA base damage (assessment of nuclear oxidized guanine (8-OHG to assess base oxidation damage and a marker of oxidative stress^41^) was significantly increased with chronic iron exposure in FT194 cells (relative to untreated) (*p* < 0.0001). In contrast, we did not observe a significant change in DNA damage in transformed FT194-OCV cells (relative to FT194-CV) for either γH2AX (*p* = 0.0821) or nuclear 8-OHG (*p* = 0.9559; Fig. 4b). In addition, we identified an increase in cytoplasmic base oxidation damage, likely representing damaged RNA^42^, in both FAC-exposed and OCV cell lines.

Since there is evidence that transition metals can promote DNA damage resulting in gene mutations^43^, we hypothesized that chronic iron exposure may be able to cause such mutations as well^44^. To address this question, we isolated genomic DNA from untreated and chronic FAC-treated FT194 cells for reverse transcriptase PCR using K-Ras specific primers, a common mutated gene in tumorigenesis, as previously described^30^. Sequencing analysis, however, did not identify a mutation at codon 12 in FAC-treated cells relative to untreated (results not shown).

**Fig. 5:**
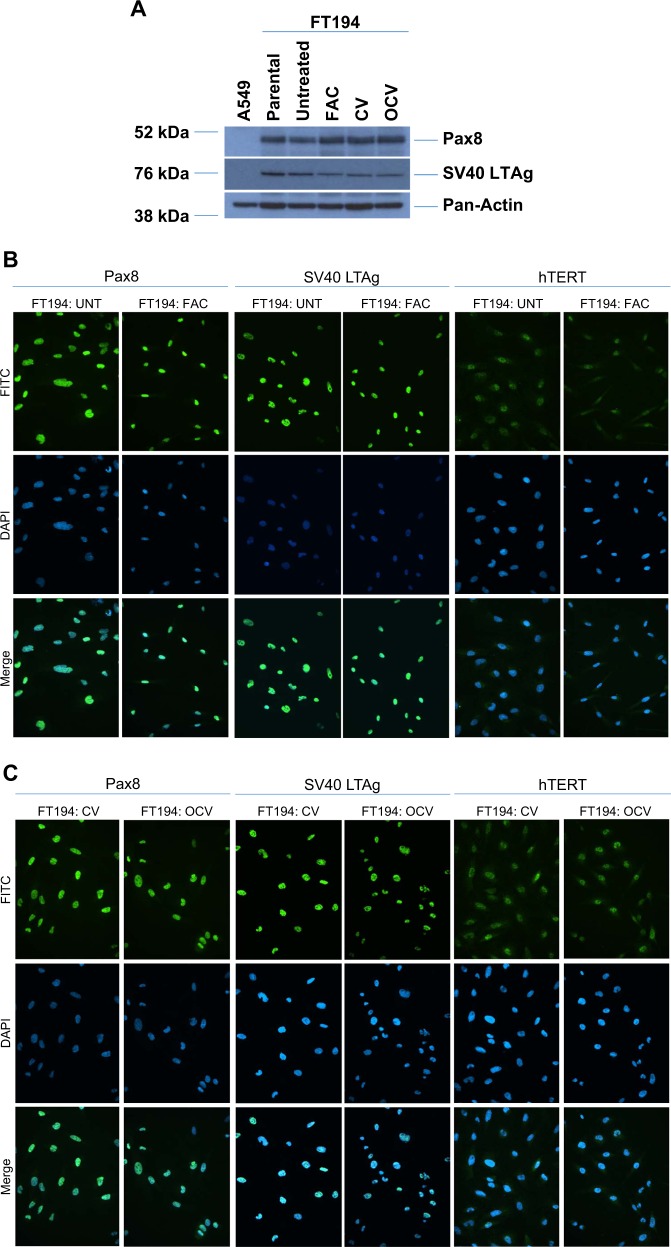
Chronic iron-exposed and transformed FTSECs retain Pax8^+^ and FoxJ1^−^ expression. **a** Lysates collected from A549 (*p* = n + 6), parental FT194 (*p* = 15), untreated and FAC-treated FT194 (*p* = 31), as well as CV and OCV FT194 (*p* = RV + 12) cells were subjected to SDS-PAGE and western analyses with the indicated antibodies. Data are representative of three independent replicates. Immunofluorescence images for Pax8, SV40 LTAg, and hTERT in FT194 untreated and FAC-treated (**b**; *p* = 24–29, days 80–90) as well as FT194-CV and FT194-OCV cells (**c**; *p* = RV + 7) from three independent experiments are shown. Images were captured at ×20 magnification

To ensure that the changes noted above occurred in cells that maintained the parental FT194 characteristics^8,32^ (see Fig. 1), we validated that the expression levels of Pax8, FoxJ1, SV40 LTAg, and hTERT were unaltered via western blotting and immunofluorescence (Fig. 5a–c). Indeed, neither chronic iron exposure nor cellular transformation altered the expression of Pax8 (100% positive), SV40 LTAg (~98–99% positive) and hTERT (Fig. 5b, c). Furthermore, FoxJ1 was not detected in any FTSEC cell line although A549 cells were weakly positive for this ciliated marker (results not shown) while negative for Pax8. Collectively, these results show that the proportion of Pax8^+^/FoxJ1^−^ cells was not altered with chronic iron exposure or oncogenic transformation and further, long-term iron exposure increased the levels of oxidative RNA and DNA damage in the absence of K-Ras mutations.

### Characterization of chronic iron-treated and transformed FTSECs reveals altered expression of EVI1 variants

**Fig. 6:**
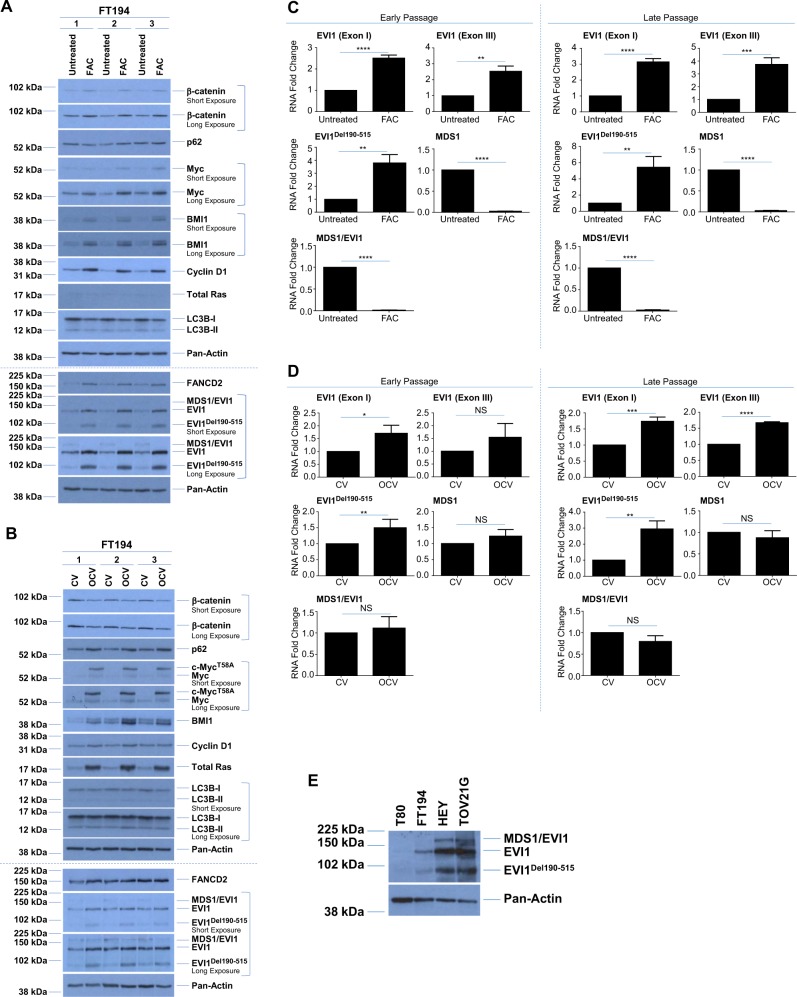

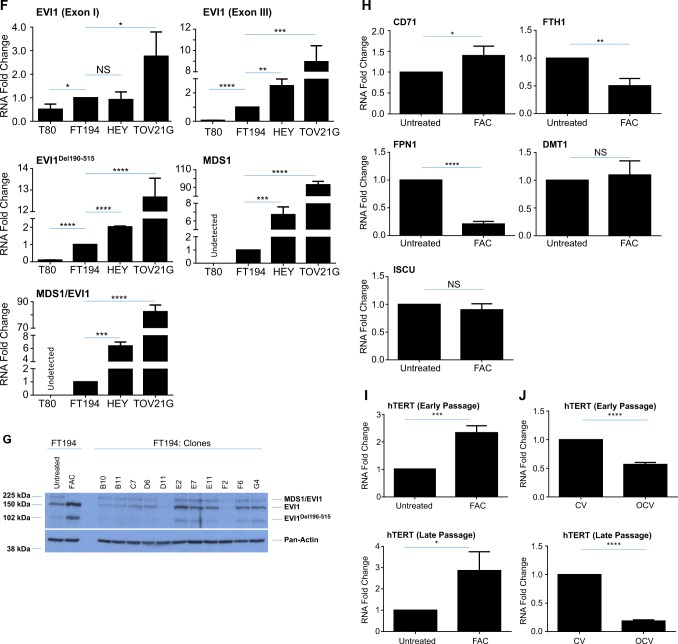
Altered expression of EVI1 variants with chronic iron exposure and cellular transformation. Western blot analyses were completed using lysates from the untreated and FAC-treated (**a**; *p* = 28, day 99) as well as CV and OCV (**b**; *p* = RV + 9) FT194 cells with the indicated antibodies. Three technical replicates are shown and the dotted line indicates the same samples run on 10% (top) or 8% (bottom) SDS-PAGE gels. RNA was isolated from **c** untreated and FAC-treated early (*p* = 20, day 69) and late (*p* = 31, day 104) passage as well as **d** CV and OCV early (*p* = RV + 3) and late (*p* = RV + 12) passage FT194 cells to assess mRNA expression of the EVI1 splice variants via real-time PCR. Data represent three independent experiments. **e** Western blot analyses using lysates from gynecological cell lines (T80, FT194, HEY, and TOV21G) are presented; samples were originally collected for analysis in our prior published work^36^, but rerun on a 8% SDS-PAGE gel to assess EVI1 variants. Data represent three independent replicates. **f** RNA was isolated from gynecological cell lines, originally collected for analysis in our prior published work^36^, but reanalyzed by normalizing to cyclophilin A (PPIA) for real-time PCR analysis of EVI1 variants. Data represent three independent replicates. **g** Protein lysates collected from clones derived from parental FT194 cells were assessed via western blot analyses with the indicated antibodies and compared with untreated and FAC-treated FT194 cells (samples from Fig. 6A were rerun for comparison). **h** RNA was isolated from untreated and FAC-treated (*p* = 20, day 69) to assess mRNA expression of iron regulatory markers. Data are representative of three independent replicates. RNA was isolated from untreated and FAC-treated **i** early (*p* = 20, day 69) and late (*p* = 31, day 104) as well as CV and OCV **j** early (*p* = RV + 3) and late (*p* = RV + 12) FT194 cells to assess hTERT mRNA expression via real-time PCR. Data represent three independent experiments

Since evidence in myeloid leukemias implicates N-Ras activation in positively regulating EVI1 expression^45^ and since EVI1 (located at chromosome 3q26.2) lies within a locus commonly amplified in HGSOC^7,9^, we next investigated whether overexpression of H-Ras in the transformed FT194 cells could modulate EVI1 protein levels. Interestingly, we found moderately increased expression of wild-type EVI1 (~1.8-fold, *p* = 0.0019) and EVI1^Del190–515^ (~3.8-fold, *p* = 0.0028, also referred to as Δ324^46^) with reduced MDS1/EVI1 (~72% reduction, *p* = 0.0286) in transformed FT194 cells relative to controls (Fig. 6b). In support, as shown in Supplementary Fig. 3a, wild-type EVI1 and EVI1^Del190–515^ were also markedly elevated following H-Ras overexpression in T80 cells and similar increases in these EVI1 variants were observed in transformed human endometriotic cells (transformed via stable expression of H-Ras^V12A^ and c-Myc^T58V^, see^36^; Supplementary Fig. 3b). Although we did not observe a change in Ras protein following chronic FAC exposure (relative to untreated), we did identify a marked increase in the protein expression of wild-type EVI1 (~2.7-fold, *p* = 0.0011) and the EVI1^Del190–515^ variant (~7.3-fold, p = 0.0003), with reduced expression of MDS1/EVI1 (~76% reduction, *p* = 0.0059) (Fig. 6a). Indeed, it is remarkable that elevated expression levels of wild-type EVI1 and EVI1^Del190–515^ were previously reported to be associated with a poor patient outcome whereas elevated expression of MDS1/EVI1 was associated with an improved patient outcome^9^.

To provide support for the protein level alterations, we next assessed the mRNA expression of these EVI1 variants via real-time PCR (using custom Taqman probes and primers, as previously reported^9,47^). As shown in Fig. 6c (left panels, following ~70 treatment days), we observed ~2.5-fold increase in EVI1 Exon I (specific to wild-type EVI1, *p* < 0.0001), ~2.5-fold increase in EVI1 Exon III (specific to both EVI1 and MDS1/EVI1 variants, *p* = 0.0011), and ~fourfold increase in EVI1^Del190–515^ (*p* = 0.0016) in FAC-treated cells relative to untreated, whereas MDS1 (specific to MDS1, *p* < 0.0001) and MDS1/EVI1 (specific to MDS1/EVI1, *p* < 0.0001) were significantly (~98%) reduced in chronic iron-exposed FT194. Furthermore, prolonged maintenance of these cells (~104 treatment days) showed that these changes in expression were not only sustained but also more pronounced (Fig. 6c, right panels), with ~threefold increase in EVI1 Exon I (*p* < 0.0001), ~fourfold increase in EVI1 Exon III (*p* = 0.0008), and ~fivefold increase in EVI1^Del190–515^ (*p* = 0.0045). In transformed FTSECs, EVI1 variants were also altered in a similar pattern which was exacerbated following prolonged culturing (in late passage, *p* = RV + 12) (Fig. 6d, right panels) relative to early passage (*p* = RV + 3; Fig. 6f, left panels). Specifically, we identified increased expression of EVI1 Exon I (~1.7-fold, *p* = 0.0007), EVI1 Exon III (~1.7-fold, *p* < 0.0001), and EVI1^Del190–515^ (~threefold, *p* = 0.0026) as well as reduced expression of MDS1 and MDS1/EVI1 variants though these changes were not significant (*p* = 0.2579 and *p* = 0.0576, respectively). Altogether, these gene expression changes implicate chronic iron exposure and c-Myc/H-Ras activation in the regulation of EVI1 variant expression.

Since precursors to HGSOC are postulated to arise from either the OSE or FTE^5,6,48^, we thus next investigated whether the expression of EVI1 variants differed between T80 ovarian surface epithelial cells (which did not show increased cell numbers with chronic FAC treatment, see Supplementary Fig. 1) and FTSECs. Indeed, our prior research showed that T80 cells have low expression of wild-type EVI1 and no expression of EVI1^Del190–515^ or MDS1/EVI1^9^. As presented in Fig. 6e, western blot analyses comparing T80 and FTSEC, among other ovarian tumor cell lines (namely, HEY and TOV21G epithelial ovarian carcinoma), show that T80 cells had significantly lower expression of wild-type EVI1 and EVI1^Del190–515^ with undetectable MDS1 or MDS1/EVI1 expression relative to FT194. The highest expression of these variants was observed in the two OVCA cell lines, and these protein patterns were also consistent at the mRNA level (Fig. 6f).

To address whether chronic iron exposure could select for cells better able to grow under stress and thus may be promoting growth of cells expressing high levels of EVI1 variants, we completed clonal isolation through single-cell seeding of untreated FT194 cells. Western analysis of these cell clones identified that the ratio between MDS1/EVI1, wild-type EVI1 and EVI1^Del190–515^ in the clonally isolated cell lines did not differ to that from the parental population and thus was dissimilar to that noted in the FAC-exposed cells (Fig. 6g). These results suggest that the altered expression of EVI1 variants following chronic iron exposure is unlikely due to the selection of cells expressing an elevated profile of EVI1 variants.

### Altered expression of key tumorigenic modulators in chronic iron-exposed and transformed FTSECs

We next characterized the protein expression of other common oncogenes characteristic of HGSOC in both chronic iron-exposed and transformed FT194 cells; specifically, we examined c-Myc (amplified at 8q24.21 in over 20% of OVCA patients^7^), β-catenin (which is correlated with poor patient prognosis in OVCA^49^), Cyclin D1 (a cell cycle mediator whose expression is regulated by β-catenin and is also associated with poor patient outcome^49,50^), and B cell-specific Moloney murine leukemia virus integration site 1 (BMI1; purported to have a role in the pathogenesis of OVCA^51^). As shown in Fig. 6a, we observed a marked increase in the protein expression of all of these oncogenic markers in FAC-treated cells relative to untreated. For example, in chronic FAC-treated FT194 cells, β-catenin was elevated ~2.3-fold (*p* = 0.0134) whereas Myc was elevated ~2.4-fold (*p* = 0.0040). We also noted a marked increase in the expression of FANCD2, a protein that regulates replication fork integrity and DNA repair^52^. This may be another indicator of increased cellular proliferation caused by FAC, which would require more robust support for replication fork integrity maintenance. It is interesting that cells from patients with Fanconi Anemia (FA, a rare disorder characterized by defects in DNA damage repair) harbor genomic amplification of EVI1^53^, thus implicating a potential link between FANCD2 and EVI1 (see Supplementary Fig. 3b). Also, a significant increase of Cyclin D1 was noted, which is characteristic of active proliferation, in addition to elevated BMI1, also associated with tumorigenesis^51^. Please see Supplementary Table 1 for densitometric and statistical analyses. These results were confirmed in two independent FT194 cell lines maintained up to 94 days with 250 nM FAC (Supplementary Fig. 4). Altogether, these data suggest that chronic iron treatment of FTSECs may promote the expression of oncogenes associated with HGSOC as well as markers involved in DNA repair. We also assessed the expression of the above described markers in FT194-CV and FT194-OCV cells. Exogenous mutant c-Myc protein was markedly elevated (~17.2-fold, *p* < 0.0001) in OCV relative to CV cells (as expected, Fig. 6b). Note that c-Myc^T58A^ contains the electronegative FLAG tag on its N-terminus, which results in a slower mobility on SDS-PAGE relative to endogenous Myc. In contrast to the FAC-treated FT194 cells (relative to untreated), β-catenin was reduced (by ~31.8%, *p* = 0.0544). Moreover, BMI1 protein was increased consistently, FANCD2 was unchanged, and cyclin D1 was only subtly elevated in FT194-OCV (relative to FT194-CV). With respect to FANCD2, its expression could be a result of increased oxidative damage caused by iron exposure similar to prior findings^54^. These results indicate that there are alterations in the expression of key oncogenes following chronic FAC exposure but these changes differed to some extent with transformation.

Since prior research implicates altered iron regulation in immortalized and transformed fallopian tube precursors^55^, we characterized the mRNA expression of the key iron pathway mediators in our chronic iron-exposed FTSECs. Indeed, we identified a significant increase in the expression of transferrin receptor (CD71, *p* = 0.0360) concurrent with significantly reduced expression of the iron storage protein ferritin (FTH1, *p* = 0.0028) and the iron exporter ferroportin (FPN1, *p* < 0.0001), with no observed changes in the mRNA expression of the divalent metal transporter (DMT1, *p* = 0.5492) or the iron–sulfur cluster assembly protein (ISCU, *p* = 0.1989; see Fig. 6h). These observations suggest that there may be increased intracellular iron retention in FTSECs following chronic iron exposure.

Since we noted that Myc was elevated following chronic iron exposure of FTSECs, we next addressed whether hTERT expression was altered (hTERT is involved in telomere maintenance, is commonly overexpressed in cancer^56^, and is regulated transcriptionally by c-Myc^57^). As shown in Fig. 6i, we identified an approximately two–threefold increase in hTERT mRNA expression in FAC-treated cells (relative to untreated) (early passage *p* = 0.010 (top) and late passage *p* = 0.0220 (bottom)). Surprisingly, there was a significant reduction (up to 80%) in hTERT transcripts in FT194-OCV relative to FT194-CV (*p* < 0.0001, Fig. 6j); and furthermore, the change in hTERT expression was exacerbated in both late passage FAC and OCV cell lines (relative to their controls). These results uncover alterations in hTERT mRNA expression with both chronic iron treatment and in transformed FTSECs.

### EVI1 variants, β-catenin, and FANCD2 are regulated in an autophagy-independent and proteasome-dependent manner in chronic iron-treated FT194 cells

**Fig. 7:**
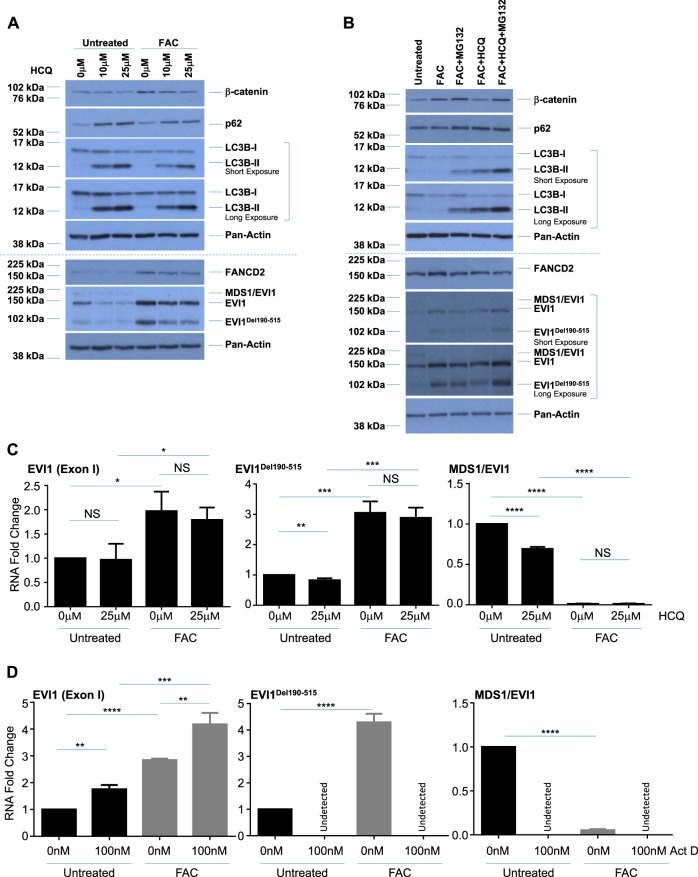

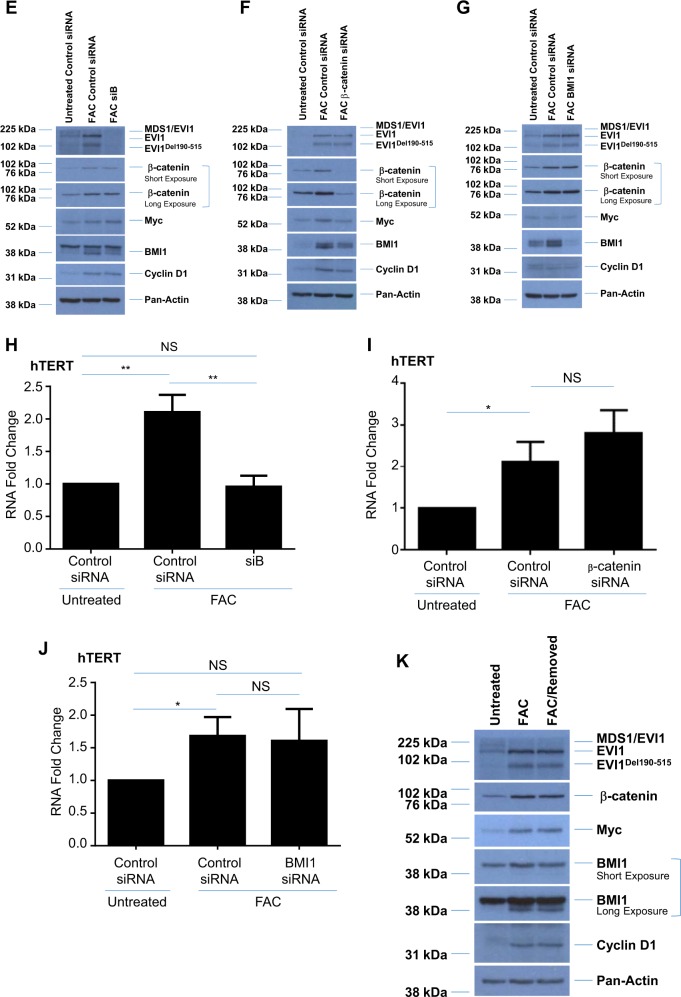
EVI1 variants are regulated in an autophagy-independent and β-catenin-independent manner in chronic iron-exposed FT194 cells. **a** Untreated and FAC-treated FT194 cells (*p* = 23, day 78) were treated with 10 μM or 25 μM HCQ for 18 h. Lysates were collected and subjected to western blot analyses with the indicated antibodies. The dotted line indicates the same samples run on 10% (top) or 8% (bottom) SDS-PAGE gels, and data are representative of four independent replicates. **b** Untreated and FAC-treated FT194 cells (*p* = 28, day 99) were treated with 25 μM HCQ and/or 5 μM MG132 for 18 h, and western blot analyses were completed with the indicated antibodies. The dotted line indicates the same samples run on 10% (top) or 8% (bottom) SDS-PAGE gels, and data are representative of three independent replicates. RNA was isolated from untreated and FAC-treated FT194 cells following (**c**; *p* = 26, day 92) 18 h treatment with 25 μM HCQ, or (**d**; *p* = 39, day 136) 18 h treatment with 100 nM Act D, to assess the indicated EVI1 splice variants via real-time PCR. Data represent three independent experiments. Western blot analyses were completed with the indicated antibodies following siRNA knockdown of EVI1 (siB, **e**; *p* = 32, day 113), β-catenin (**f**; *p* = 36, day 124), and BMI1 (**g**; *p* = 29–30, day 102–104). Data are representative of three independent experiments. RNA was isolated from untreated and FAC-treated FT194 cells following transfection with siRNAs targeting EVI1, siB (**h**; *p* = 27, day 86), β-catenin (**i**; *p* = 24, day 81), and BMI1 (**j**; *p* = 31, day 109), to assess hTERT mRNA expression (relative to respective control nontargeting siRNA in both untreated and FAC-treated cells) via real-time PCR. Data represent three independent replicates. **k** FAC-treated FT194 cells (*p* = 34, day 120) were maintained in the absence of 250 nM FAC for 4 days, as indicated in the “Materials and methods” section. Western blot analyses were completed with the indicated antibodies and data are representative of three independent experiments

Since we previously reported that the autophagic pathway was altered in response to acute iron overload conditions in gynecological cells^30^ and that EVI1 expression may regulate the expression of PDZ-binding kinase which leads to increased autophagy to confer chemoresistance^58^, we next investigated whether chronic iron exposure may also alter the autophagic pathway. We identified a subtle reduction in both LC3B-I and LC3B-II (the nonconjugated and conjugated forms, respectively) protein without a clear notable change in p62/sequestosome protein (Fig. 6a), suggesting that autophagic flux may be altered in cells following chronic iron exposure (relative to untreated cells). To identify whether autophagic flux is altered under these conditions, we treated untreated and chronic FAC-treated FT194 cells with 10 µM or 25 µM hydroxychloroquine (HCQ) for 18 h to inhibit autophagic flux^59,60^. We found that LC3B and p62 accumulated with HCQ treatment in both untreated and FAC-treated cells, suggesting that initiation of the autophagic pathway was still active in both cell lines (as a reduction in autophagy initiation would show no change in LC3 and p62 expression with HCQ treatment) (Fig. 7a). We also found that EVI1^Del190–515^, FANCD2, and β-catenin were reduced following treatment with HCQ independent of iron treatment, suggesting an alternative mechanism of protein regulation. Indeed, cotreating FAC-exposed FT194 cells with both HCQ and MG132 (a proteasome inhibitor^61^) recovered wild-type EVI1 and EVI1^Del190–515^ protein expression, as well as FANCD2 and β-catenin expression, relative to HCQ alone (Fig. 7b), suggesting that these markers are regulated in a proteasome-dependent manner. Please see Supplementary Table 1 for densitometric and statistical analyses.

To determine whether targeting autophagy may alter mRNA expression of the EVI1 variants, we next performed real-time PCR following treatment with HCQ. While there was no significant reduction of wild-type EVI1 mRNA in either untreated (*p* = 0.8732) or FAC-treated (*p* = 0.5491) cells, we observed significantly reduced EVI1^Del190–515^ mRNA with HCQ in untreated (*p* = 0.0098) but not FAC-treated (*p* = 0.5981) cells (Fig. 7c). Furthermore, there was a significant reduction of MDS1/EVI1 mRNA with HCQ in untreated cells (*p* < 0.0001) but not in FAC-treated cells (*p* = 0.8922), suggesting that the protein expression of these EVI1 splice variants is regulated by autophagy in untreated cells, which is lacking with chronic iron exposure. Since we observed a significant reduction in the mRNA levels of EVI1^Del190–515^ and MDS1/EVI1 with HCQ, we next determined whether transcriptional inhibition with Actinomycin D (Act D)^62^ also altered mRNA transcripts of EVI1. Indeed, as shown in Fig. 7d, transcriptional inhibition reduced the expression of EVI1^Del190–515^ and MDS1/EVI1 variants to undetectable levels in both untreated and FAC-treated cells, while wild-type EVI1 mRNA was significantly increased in untreated (*p* = 0.0014) and FAC-treated (*p* = 0.0059) FT194 cells relative to controls. Together, these results suggest that inhibiting transcription increases the stability of wild-type EVI1 mRNA and provides novel insight into the regulation and expression of other EVI1 variants.

### Knockdown of EVI1 variants reduces hTERT mRNA whereas β-catenin and BMI1 knockdown reduce Myc and Cyclin D1 proteins

Since the protein expression of oncogenic EVI1 variants, BMI1, and β-catenin were dramatically elevated following chronic iron exposure in FT194 cells (see Fig. 6a), we next investigated the effect of knocking down each marker in FAC-treated cells on downstream targets. As shown in Fig. 7e, siRNA-mediated knockdown of all EVI1 variants with siB^46,47^ did not alter the expression of β-catenin, Myc, or BMI1. Knocking down β-catenin resulted in a reduction of Myc, Cyclin D1, and BMI1 protein levels in FAC-treated FT194 cells without affecting the protein expression of the EVI1 splice variants (Fig. 7f), whereas knocking down BMI1 showed a possible reduction in β-catenin, but no marked change in Cyclin D1, Myc or EVI1 protein expression relative to control (Fig. 7g).

Since we observed both elevated hTERT mRNA and EVI1 expression with chronic FAC treatment, and since there is evidence for a possible link between EVI1 and hTERC (the RNA component of hTERT) amplification in non-small cell lung cancers^63^, we next determined whether hTERT mRNA expression was altered with knockdown of EVI1. Interestingly, siB-transfected FAC-treated FT194 cells showed a significant reduction in hTERT mRNA relative to control nontargeting siRNA (*p* = 0.0032) (Fig. 7h). In contrast, there was no reversal of hTERT mRNA following β-catenin siRNA or BMI1 knockdown in FAC-treated cells (*p* = 0.1773 (Fig. 7i) and *p* = 0.8309 (Fig. 7j)). These results indicate that hTERT may be transcriptionally regulated via EVI1 variants.

We next questioned whether maintaining FAC-treated FT194 cells in the absence of iron would reverse the expression of key oncogenic markers. However, as shown in Fig. 7k, maintaining chronic FAC-treated FT194 cells for 4 days without FAC-supplemented media elicited no change in the protein expression of the EVI1 variants, Myc, β-catenin, BMI1, or Cyclin D1. Thus, these results suggest that chronic iron treatment of FTSECs may have caused permanent changes (i.e., genomic or epigenetic).

## Discussion

Recent evidence suggests that iron may contribute to OVCA pathogenesis. Indeed, NTBI levels are elevated in patients with hereditary hemochromatosis and have been linked to the development of HGSOC^21,24^. Iron deposits have also been identified in the fallopian tubes of patients with HGSOC^27^, implicating elevated levels of iron at locations where the proposed precursors to HGSOC have been identified^29^. Further, tubal ligation (which prevents retrograde menstruation, in turn reducing heme and transferrin accumulation in the peritoneal cavity) is associated with a decreased risk of OVCA; indeed, salpingectomy results in the absence of OVCA and any consequent metastatic OVCA^64^. Although exposing primary FTSECs to catalytic sources of iron increased their proliferative capacity^26^, a causative relationship between iron exposure and increased OVCA risk has not yet been clearly identified. However, a thorough experimental investigation of the mechanism of action of chronic iron exposure is needed.

**Fig. 8:**
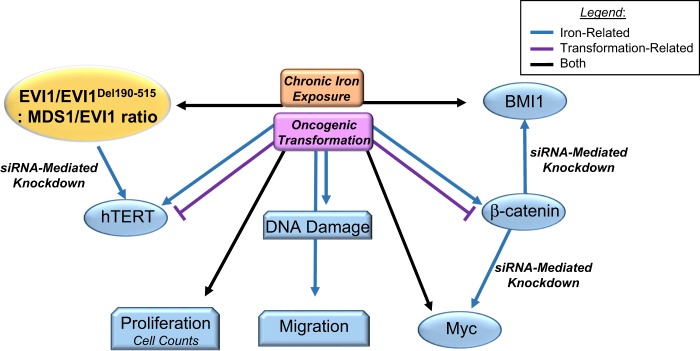
Model of FAC-induced pathogenesis of FTSECs compared with oncogenically transformed FTSECs. FAC-exposed FTSECs (for >2 months) demonstrated increased migration in addition to increased DNA damage/repair marker expression such as FANCD2. Furthermore, these cells had elevated hTERT mRNA. Transformed cells (as well as the iron-exposed cells) elicited increased cell numbers and expression of EVI1 variants, Myc, BMI1, and Cyclin D1. EVI1 knockdown markedly reduced hTERT transcript levels. β-catenin and BMI1 knockdown diminished c-Myc and Cyclin D1 proteins

As summarized in Fig. 8, we show that chronic iron (presented as FAC (NTBI)) exposure of FTSECs results in increased DNA damage coincident with increased cell numbers and migratory potential relative to untreated FTSECs. Interestingly, these changes were associated with increased expression of oncogenic markers such as EVI1, EVI1^Del190–515^, hTERT, Myc, β-catenin, BMI1, and Cyclin D1. As increased activities of the Fanconi Anemia pathway is associated with a subpopulation of OVCAs^65^, the increased FANCD2 expression by FAC is a notable observation. While oncogenic transformation (mediated by H-Ras and c-Myc) also increased cell numbers with increased expression of EVI1, EVI1^Del190–515^, and BMI1, these transformed cells failed to display alterations in DNA damage and surprisingly, elicited a reduction in cellular migration as well as reduced expression of hTERT and β-catenin. These results demonstrate that chronic iron exposure may promote the transition of fallopian tube precursors to OVCA through mechanisms which differ from those mediated by H-Ras/c-Myc-induced cellular transformation.

Our results also potentially suggest a role for iron in promoting the transition of fallopian tube but not ovarian precursors (T80). Indeed, in contrast to FTSECs, T80 cells failed to respond to chronic NTBI treatment. Stem cell niches have been identified near both the OSE and FTE, but the contribution of each of these niches to the development of HGSOC remains unclear^66–68^. Our attempts to investigate the expression of key stem cell markers (i.e., Oct4A, Sox2, LEF1, LGR5, and ALDH1) in our chronic iron-exposed FTSECs were unsuccessful due to the lack of detection via western blotting; however, further work to address their contribution to the observed functional responses is needed.

Research shows that cellular transformation with SV40 LTAg can promote chromosomal aberrations including aneuploidy^69,70^, and we identified that FT194-CV and untreated FT194 cells contained a population of cells with >4N DNA content (aneuploid cells). It is interesting that this population of cells was similarly reduced following oncogenic transformation with H-Ras^V12A^ and c-Myc^T58A^ as well as following chronic iron exposure; we propose that these aneuploid cells may represent a population of senescent cells that are being selected against under these conditions^71,72^. Indeed, it has been shown that aneuploidy can promote senescence and reduce “cellular fitness”^73^. Furthermore, senescent cells that are able to overcome arrest have increased tumorigenic propensity and are associated with activated β-catenin signaling^74^.

Prior published work demonstrated that EVI1 is the most highly amplified gene product expressed at the 3q26.2 locus in multiple epithelial cancers^9^. In HGSOC, this region is the most common and broadly amplified region, as defined from both TCGA data^7^ and our work^9^. There exists multiple EVI1 variants which are altered following chronic iron overload; specifically, the oncogene EVI1 and EVI1^Del190–515^ were increased whereas the tumor suppressive MDS1/EVI1 form was reduced (refer to Fig. 6a). This is notable in light that the functions of these individual variants are associated with differing patient outcomes and cellular functions^47^. The mechanisms underlying the EVI1 gene expression changes are presently unclear. The removal of exogenous iron and β-catenin knockdown from the chronically exposed cells failed to reverse the expression of EVI1. This is in contrast to reports that β-catenin was able to regulate EVI1 expression^75,76^. Further investigations, such as DNA copy number changes and epigenetic profiles, would potentially uncover the mechanisms underlying these EVI1 changes.

Another important difference between chronic iron-treated and transformed FT194 cells is hTERT mRNA levels, which were significantly increased following chronic FAC exposure but significantly reduced in transformed cells (relative to their respective controls). Interestingly, we identified that EVI1 knockdown reduced hTERT mRNA expression. To our knowledge, this is the first indication that EVI1 may contribute to regulation of hTERT expression, although links between EVI1 amplification and telomerase have been previously reported^63,77^.

The proto-oncogene c-Myc, located at 8q24.21, is also commonly amplified in OVCA^7^, and was markedly elevated at the protein level with chronic iron exposure (relative to untreated FT194 cells). Upregulation of c-Myc protein could be contributed by β-catenin since its knockdown reduced Myc protein in FAC-treated cells (relative to nontargeting control siRNA; in support, β-catenin was previously reported to regulate c-Myc expression in other cancer cells^78,79^). Knocking down β-catenin also reduced BMI1 protein expression in FAC-treated FT194 cells, and it is interesting that β-catenin knockdown in colon cancer cells also resulted in reduced BMI1 expression^80^. To our knowledge, this phenomenon has not been previously described in FTSECs, and it is important to note that BMI1 was recently described as a potential OVCA biomarker^51^. It is possible that this link between BMI1 and β-catenin is important for the oncogenic changes induced by chronic iron exposure in FTSECs, and this observation warrants further investigation.

In conclusion, we have identified that chronic iron exposure of FTSECs increases cell numbers and migration, promotes DNA damage, and increases the expression of oncogenes commonly associated with HGSOC, albeit through different mechanisms from c-Myc and H-Ras induced cellular transformation. These results suggest that iron may contribute to the transition of fallopian tube epithelial cells (and unlikely to transition of OSE) to HGSOC. Further research is needed to elucidate the mechanisms underlying this transition; such studies would provide valuable insight into the development of this deadly cancer that could aid in the development of novel therapeutic strategies.

## Materials and methods

### Cell culture and treatments

Human immortalized (expressing SV40 Large T Antigen (LTAg) and hTERT) normal ovarian surface epithelial T80 cells were kindly provided by Dr Gordon Mills (MD Anderson Cancer Center, Houston, TX), while H-Ras and K-Ras overexpressing T80 cells were kindly provided by Dr Jinsong Liu (MD Anderson Cancer Center); these cells were maintained in RPMI 1640 supplemented with 8% FBS and 1% penicillin/streptomycin as previously described^30^. Human immortalized FTSECs (FT194) were kindly provided by Dr Ronald Drapkin (Department of Obstetrics and Gynecology, University of Pennsylvania, Philadelphia, PA, USA)^28^. These cells were immortalized via stable expression of SV40 LTAg and hTERT as previously described^28^ and maintained in DMEM:F12 (1:1, #15–090-CV, Corning Incorporated, Corning, NY, USA) with phenol red, supplemented with 2% Ultroser G Serum Substitute (#67042, Crescent Chemical Company, Islandia, NY, USA) and 1% penicillin-streptomycin. Human lung adenocarcinoma cells (A549, obtained from the ATCC (Manassas, VA, USA)) were maintained in DMEM:F12 (1:1) with phenol red, supplemented with 8% FBS and 1% penicillin–streptomycin. For chronic iron treatment, immortalized FTSECs were maintained in phenol red-free DMEM:F12 (1:1, #21041-025, ThermoFisher, Waltham, MA, USA) supplemented with 8% charcoal dextran-stripped FBS and 1% penicillin/streptomycin (annotated as -PR media), as previously described^36^. All cell lines were confirmed to be mycoplasma negative. Cell line authentication was performed via STR profiling (Genetica Laboratories, Cincinnati, OH, USA), as appropriate.

FAC ((a source of NTBI), #I72-500, Fisher Scientific, Pittsburgh, PA, USA) was prepared as a 50 mM stock in PBS and used at final concentrations of 25 nM, 250 nM, 2.5 µM, 25 µM, or 250 μM^30,36^. The proteasome inhibitor, MG132 (Fisher Scientific, Pittsburgh, PA, USA), was prepared as a 10 mM stock in dimethyl sulfoxide and used at a final concentration of 5 μM^81^. The transcriptional inhibitor Act D (Fisher Scientific, Pittsburgh, PA, USA) was prepared as a 1 mg/ml stock in RPMI 1640 media (diluted to 100 μg/ml in PBS) and used at a final concentration of 100 ng/ml^82^. The autophagic flux inhibitor, HCQ, (Fisher Scientific, Pittsburgh, PA, USA), was prepared as a 50 mM stock in PBS and used at a final concentration of 10 or 25 μM^83^.

### Chronic iron exposure in FTSECs

A schematic summarizing the chronic iron treatment strategy in FT194 cells is presented in Fig. 2a. These cells were initially seeded at 500 cells/well (50 cells/cm^2^) in a six-well plate. The following day, attached cells were rinsed with PBS, and media was exchanged to PR media with 0, 25 nM, 250 nM, 2.5 µM, 25 µM, or 250 μM FAC; note that ammonium citrate was previously tested as a control for FAC^23^. Cell growth was monitored closely; after 16 days of treatment, FT194 cells (at 0, 25 nM, 250 nM, 2.5 µM, and 25 μM) approached confluency and were thus independently expanded to T75 flasks in -PR media for continued treatment at the respective FAC doses. Since cells maintained in 250 nM FAC appeared to be more numerous upon continued maintenance (compared with untreated or other FAC doses), the FT194 cells undergoing 250 nM treatment were selected for prolonged maintenance in T75 flasks. Untreated cells were maintained in -PR media concurrently with the 250 nM FAC-treated cells to account for spontaneous mutations (and other potential events) that could arise from long-term culturing^84^. Upon nearing confluency (approximately every 12 days), both untreated and 250 nM FAC-treated FT194 cells were counted using a hemocytometer and reseeded at a low density of 37,500 cells (500 cells/cm^2^) into T75 flasks. Cells were functionally assessed between 2 and 4 months after initiating FAC treatment; these findings were functionally validated with two additional replicates generated in a similar manner.

### Generation of transformed FTSECs

Transformed FTSECs were developed using a methodology similar to that previously reported^36,37^. Briefly, HEK293T packaging cells, seeded at 1.5 × 10^6^ cells/well in six-well plates, were transfected with retroviral expression plasmids in combination with pCGP and pVSVG plasmids (at a 1:1:1 ratio) using Fugene HD (Promega, Madison, MI, USA). Specifically, CV was generated using an empty retroviral pBABE-puro expression plasmid (Addgene plasmid #1764^85^) whereas OCV was generated using (a) H-Ras^V12A^ (Addgene plasmid #9051, a gift from William Hahn), (b) c-Myc^T58A^ (Addgene plasmid #20076, a gift from Juan Belmonte^86^), and (c) SV40 LTAg (Addgene plasmid #14088, a gift from William Hahn^87^) retroviral expression plasmids at equimolar concentrations. After 48 h of transfection, retrovirion-containing media was collected, filtered (0.45 μm), and used to infect FT194 cells (seeded the day prior at 250,000 cells/well in a six-well plate) with 8 μg/ml polybrene. Puromycin selection up to 1 μg/ml was performed to allow for selection of cells expressing the transduced oncogenes; Fig. 2b presents a schematic describing the retroviral transduction process for FTSEC cellular transformation.

### K-Ras sequencing analyses

Genomic DNA was isolated from untreated and chronic FAC-treated FT194 cells using the DNA DNeasy Blood and Tissue kit per manufacturer’s protocol (Qiagen, Valencia, CA, USA). K-Ras primers to amplify codon 12 were used for polymerase chain reaction (PCR) as previously described^30^. The amplified PCR product was analyzed on a 1% agarose gel and DNA purified using the QIAQuick Gel Extraction Kit per manufacturer’s instructions (Qiagen, Valencia, CA, USA). DNA sequencing analysis of the purified PCR product was completed by Eurofins Genomics (Louisville, KY, USA) and the sequencing results were aligned to K-Ras (NM_004985.4) using Genomatix Dialign for analyses^88^.

### Clonogenic assay

Clonogenic Assay was completed as previously described^36^. Briefly, FT194 cells (untreated, FAC-treated, CV, and OCV) were seeded at 500 cells/cm^2^ (equivalent cell density to seeding in T75 flasks, as described in the “Chronic iron exposure in FTSECs” section above) in six-well plates and maintained in culture for 10–17 days prior to crystal violet staining. Following cell staining, 1 ml of Sorensen’s Buffer (0.1 M sodium citrate (pH 4.5) and 50% ethanol) was added to each well and incubated for 2 h on a rotating shaker to dissolve the crystal violet into solution. Samples were then pipetted into a 96-well plate (100 μl per well) and analyzed on a BioTek plate reader at 570 nm.

### Immunofluorescence

FTSECs were seeded at 250,000 cells/well in six-well plates onto glass coverslips and handled as previously described^36^. Briefly, at 2 days post seeding, cells were fixed for 30 min in 4% formaldehyde (diluted in PBS) followed by blocking for 1 h (5% goat serum and 0.1% Triton X-100 in PBS). The cells were next incubated overnight in primary antibody solution (prepared with 1% goat serum and 0.1% Triton X-100 in PBS) in a humidifying chamber at 4 °C, followed by a 1 h incubation at room temperature in either goat anti-rabbit or goat anti-mouse secondary antibody (at 1:500 prepared in 1% goat serum and 0.1% Triton X-100 in PBS) (#A11008/A11029, Alexa Fluor-488, Fisher Scientific, Pittsburgh, PA, USA)) before mounting onto glass slides with Vectashield antifade solution (#NC9524612, Fisher Scientific, Pittsburgh, PA, USA). Images were captured at both ×20 and ×63 (oil objective) using a PerkinElmer UltraVIEW Confocal spinning disc microscope (PerkinElmer Corporation). The primary antibodies used were: (1) Pax8 rabbit polyclonal (1:250, #10336-1-AP, ProteinTech, Rosemont, IL, USA), (2) FoxJ1 mouse monoclonal (1:100, #sc-365216, Santa Cruz Biotechnology, Dallas, TX, USA), (3) SV40 LTAg mouse monoclonal (1:500, #554149, BD Biosciences, San Jose, CA, USA), (4) hTERT rabbit polyclonal (1:500, #sc-7212, Santa Cruz Biotechnology, Dallas, TX, USA), and (5) γH2AX rabbit monoclonal (1:400, #9718, Cell Signaling Technology, Danvers, MA, USA).

### Assessment of DNA and RNA base damage via immunofluorescence

For assessing DNA and RNA damage, cells were seeded onto glass coverslips and processed as described in the Immunofluorescence section, with the following changes. For assessment of DNA base damage, after 4% formaldehyde fixation and blocking, samples were incubated for 1 h in RNase solution (containing 10 mM Tris-HCl, 0.1% Triton X-100, 15 mM NaCl, and 0.2 mg/ml RNase in PBS). Next, samples were incubated in 2 M HCl for 10 min at room temperature, followed by a rinse with 50 mM Tris. Next (for both DNA and RNA base damage assessment, primary antibody incubation occurred with 8-OHG mouse monoclonal (1:200, #ab62623, Abcam, Cambridge, MA, USA) followed by secondary antibody incubations. Confocal images were captured as detailed in the Immunofluorescence section, above.

### Protein isolation, SDS-PAGE, and western blotting

SDS-PAGE and western analyses were completed as previously described^89,90^. Briefly, normalized protein samples were loaded onto either 8% or 10% SDS-PAGE gels (as appropriate) and western blotting was completed with the following antibodies from Cell Signaling Technology (Danvers, MA, USA): (1) EVI1 rabbit monoclonal (#2593, 1:500), (2) Myc rabbit monoclonal (#13987, 1:500), (3) total Ras rabbit monoclonal (#3339, 1:1000), (4) β-catenin rabbit polyclonal (#9587, 1:1000), (5) BMI1 rabbit monoclonal (#6964, 1:1000), (6) LC3B rabbit polyclonal (#2775, 1:1000), and (7) Pan-Actin rabbit polyclonal (#4968, 1:500). SV40 LTAg mouse monoclonal (#554149, 1:1000) and p62 mouse monoclonal (#610832, 1:1000) antibodies were obtained from BD Biosciences (San Jose, CA, USA). FANCD2 mouse monoclonal (#sc-20022, 1:1000), Cyclin D1 rabbit polyclonal (#sc-718, 1:1000), and FoxJ1 mouse monoclonal (#sc-365216, 1:500) were obtained from Santa Cruz Biotechnology.

### Cell cycle analyses

Cells were seeded at 250,000 cells/well in six-well plates and grown for 2 days. Both nonadherent and adherent cells were collected and fixed for 1 h using ice-cold 70% ethanol (in PBS). After two PBS washes, cell pellets were suspended in propidium iodide stain solution consisting of 0.1% Triton X-100, 0.02 mg/ml propidium iodide, and 0.2 mg/ml RNase (prepared in PBS) and incubated at room temperature for 30 min. Samples were filtered (using a 0.4 μm nylon mesh) to remove aggregates and then analyzed using a BD Accuri C6 Flow Cytometer (BD Biosciences, San Jose, CA, USA). Gates were set using an unstained control sample.

### Boyden chamber cell migration assay

Cell migration was completed as per manufacturer’s protocol (#CBA-100, Cell Biolabs, San Diego, CA, USA) and as previously described^36^. Briefly, 30,000 FT194 cells (untreated, chronic iron-treated, CV, or OCV) were seeded in respective serum-free media into Boyden Chamber inserts in a 24-well plate, with respective complete media added to the lower chamber. For chronic iron-treated FT194 cells, 250 nM FAC was included in both the insert (with the cells) and the lower chamber. Cells remained in culture for 24 h, after which the migrated cells were stained with cell stain solution. Light microscope images were captured at ×100 magnification and the migrated cells counted.

### RNA isolation and real-time PCR

The RNeasy Kit (QIAGEN, Valencia, CA, USA) was utilized for RNA isolation following the manufacturer’s instructions, as previously reported^30,36^. Real-time PCR was performed using the TaqMan™ RNA-to-CT™ 1-Step Kit from ThermoFisher (#4392938, Waltham, MA, USA)^30^. The following custom-designed FAM-labeled probes/primers were used: (1) EVI1 Exon I (specific for EVI1), (2) EVI1 Exon III (specific for EVI1 and MDS1/EVI1), (3) EVI1^Del190–515^, (4) MDS1, (5) MDS1/EVI1 (see^9,47^ for primer and probe sequences), and (6) custom-designed hTERT:

Forward primer: 5′-CGCAGGGCTCCATCCT-3′

Reverse primer: 5′-TCCCCGCAAACAGCTTGT-3′

Probe sequence: 5′-CTCTGCAGCCTGTGCTAC-3′

*C*_*T*_ values were normalized to either β-actin (#401846, Applied Biosystems, Foster City, CA, USA) or Cyclophilin A (PPIA, #Hs04194521_s1, ThermoFisher, Waltham, MA, USA) as appropriate. RNA fold changes were calculated using the correlative 2^−ΔΔCT^ method.

### siRNA-mediated knockdown in FTSECs

siRNA transfections were completed as previously described^36,90^, with the following modifications. Briefly, FTSECs were seeded at 350,000 cells/well in six-well plates and allowed to adhere overnight. The following day, cells were transfected with the respective siRNA using RNAiMAX (#13778-075, Invitrogen, Carlsbad, CA, USA). Nontargeting control (#D-001810-10-20), β-catenin (#L-003482-00), and EVI1 (siB, custom designed as detailed in ref. ^46^) ON-TARGETplus siRNAs were obtained from GE Dharmacon (Lafayette, CO, USA). For BMI1 knockdown, control siRNA was obtained from Bioneer (#SN-1003) and BMI1 siRNA was obtained from QIAGEN (#SI05044473); one round of siRNA transfections was performed for all siRNA knockdown experiments described herein.

### Statistical analyses

GraphPad version 6.04 Prism software (GraphPad, La Jolla, CA, USA) was used to complete all statistical analyses; *p*-values were calculated using the nonparametric Student’s *t*-test and all error bars depict the mean ± standard deviation of at least three independent experiments, unless otherwise stated. NS represents nonsignificant *p*-values; *p-values ≤ 0.05; **p-values ≤ 0.01; ***p-values ≤ 0.001; and ****p-values ≤ 0.0001. Any indicated fold changes or percent reductions were calculated as an average of three independent replicates.

## Supplementary information

Supplementary Figure Legends.

Supplementary Figure 1.

Supplementary Figure 2.

Supplementary Figure 3.

Supplementary Figure 4.

Supplementary Table 1.
