# Supplementary material for: Chronic iron exposure and c-Myc/H-ras-mediated transformation in fallopian tube cells alter the expression of EVI1, amplified at 3q26.2 in ovarian cancer
Source: Oncogenesis. 2019 Aug 21;8(9):46. doi: 10.1038/s41389-019-0154-y (PMC6704182; doi:10.1038/s41389-019-0154-y)
Supplement: Supplementary file 1 — Supplementary Figure Legends. [file 41389_2019_154_MOESM1_ESM.docx]

**SUPPLEMENTARY INFORMATION**

**Supplementary Fig. 1 Chronic iron exposure in T80 cells does not alter cell numbers.**

Cell counts were obtained from T80 cells treated with 250 μM FAC over a period of ~200 days. Cell counts from untreated and FAC-treated cells on days 47, 67, 91, and 139 are presented.

**Supplementary Fig. 2 Clonogenic growth is elevated in chronic iron exposed and transformed FTSECs.**

**a** FT194 untreated and FAC-treated cells were seeded at 500 cells/cm^2^ (p=31) and stained 17 days post-seeding (113 days). Three independent replicates are shown. **b** FT194-CV and FT194-OCV cells were seeded at 500 cells/cm^2^ (p=RV+6) and stained 10 days post-seeding. Three independent replicates are shown.

**Supplementary Fig. 3 Analysis of EVI1 protein expression in transformed T80 and endometriotic cell lines.**

**a** Western blot analyses were completed using lysates collected from T80, H-Ras overexpressing T80, and K-Ras overexpressing T80 cells and analyzed for EVI1 expression. **b** Western blot analyses were completed for PE-A and PE-B CV/OCV cell lines, for which lysates were analyzed in our prior published work [36] and re-run on 8% SDS-PAGE gel to assess EVI1 expression.

**Supplementary Fig. 4 Validation of key oncogenic markers following chronic iron exposure.**

Western blot analyses were completed for two independent replicates of FT194 cells, chronically treated with 250 nM FAC, for the indicated proteins.

**Supplementary Table 1**

Summary of fold changes and p-values for western blots presented in **a** Fig. 6a, **b** Fig. 6b, **c** Fig. 7a, and **d** Fig. 7b.
