# Supplementary figures and images for: Chronic iron exposure and c-Myc/H-ras-mediated transformation in fallopian tube cells alter the expression of EVI1, amplified at 3q26.2 in ovarian cancer
Source: Oncogenesis. 2019 Aug 21;8(9):46. doi: 10.1038/s41389-019-0154-y (PMC6704182; doi:10.1038/s41389-019-0154-y)

Supplementary Figure 1

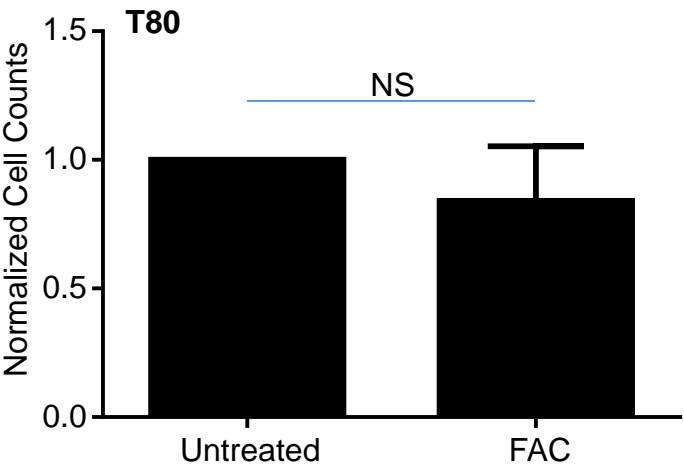

Supplement: Supplementary file 2 — Supplementary Figure 1. [file 41389_2019_154_MOESM2_ESM.pdf]

Supplementary Figure 2

A

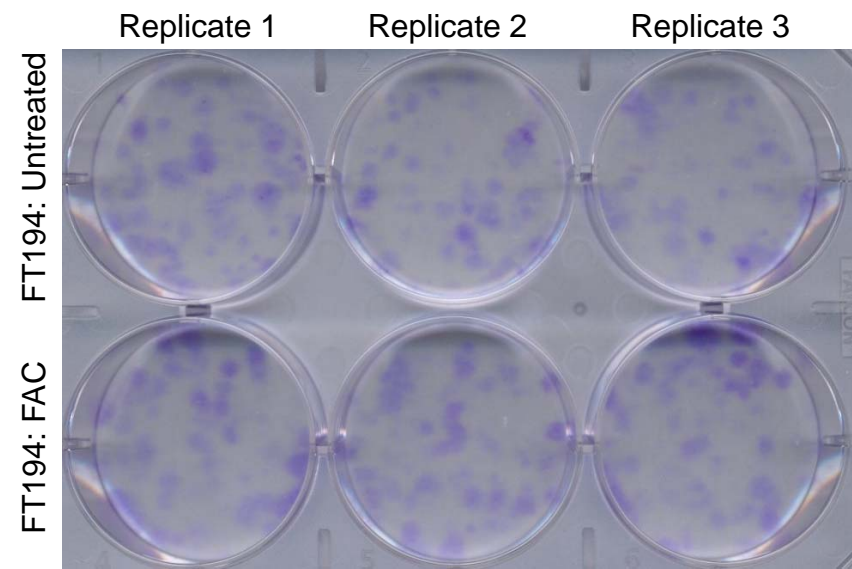

B

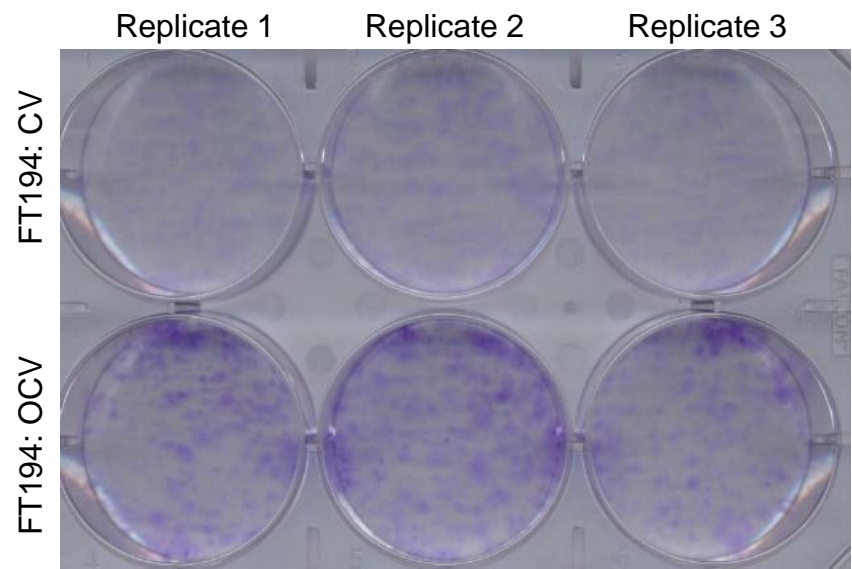

Supplement: Supplementary file 3 — Supplementary Figure 2. [file 41389_2019_154_MOESM3_ESM.pdf]

Supplementary Figure 3

A

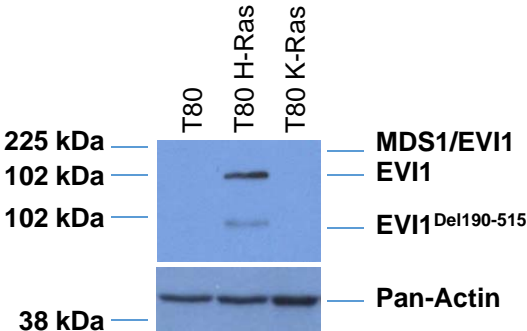

B

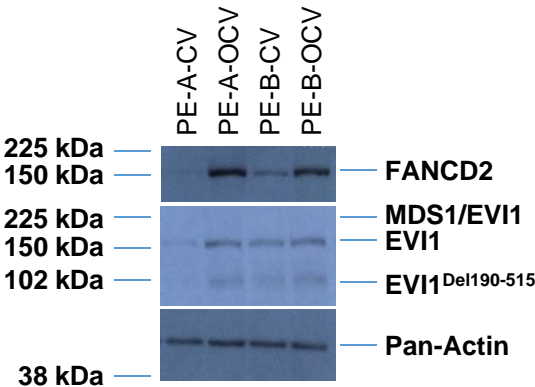

Supplement: Supplementary file 4 — Supplementary Figure 3. [file 41389_2019_154_MOESM4_ESM.pdf]

Supplementary Figure 4

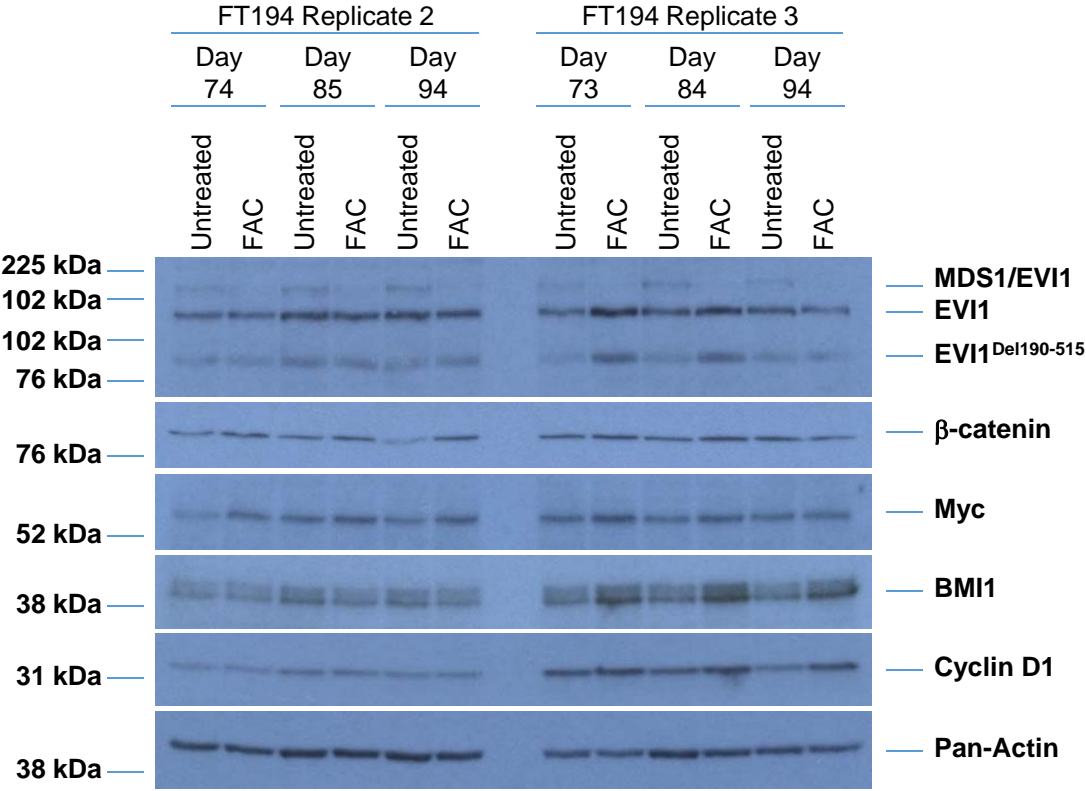

Supplement: Supplementary file 5 — Supplementary Figure 4. [file 41389_2019_154_MOESM5_ESM.pdf]
