# Supplementary material for: Chronic iron exposure and c-Myc/H-ras-mediated transformation in fallopian tube cells alter the expression of EVI1, amplified at 3q26.2 in ovarian cancer
Source: Oncogenesis. 2019 Aug 21;8(9):46. doi: 10.1038/s41389-019-0154-y (PMC6704182; doi:10.1038/s41389-019-0154-y)
Supplement: Supplementary file 6 — Supplementary Table 1. [file 41389_2019_154_MOESM6_ESM.pdf]

# Supplementary Table 1

## A

| Samples | β-catenin | | Myc | | MDS1/EVI1 | | EVI1 Wild-Type | | $EVI1^{Del190-515}$ | |
|---|---|---|---|---|---|---|---|---|---|---|
| | *Fold Change* | *p-value* | *Fold Change* | *p-value* | *Fold Change* | *p-value* | *Fold Change* | *p-value* | *Fold Change* | *p-value* |
| **Untreated** | 1.0 | - | 1.0 | - | 1.0 | - | 1.0 | - | 1.0 | - |
| **FAC** | 2.3 | 0.0134 | 2.4 | 0.0040 | 0.2 | 0.0059 | 2.7 | 0.0011 | 7.3 | 0.0003 |

## B

| Samples | β-catenin | | Myc | | MDS1/EVI1 | | EVI1 Wild-Type | | $EVI1^{Del190-515}$ | |
|---|---|---|---|---|---|---|---|---|---|---|
| | *Fold Change* | *p-value* | *Fold Change* | *p-value* | *Fold Change* | *p-value* | *Fold Change* | *p-value* | *Fold Change* | *p-value* |
| **CV** | 1.0 | - | 1.0 | - | 1.0 | - | 1.0 | - | 1.0 | - |
| **OCV** | 0.7 | 0.0544 | 17.2 | < 0.0001 | 0.3 | 0.0286 | 1.8 | 0.0019 | 3.8 | 0.0028 |

## C

| Samples | β-catenin | | p62 | | LC3B-I | | LC3B-II | | FANCD2 | | EVI1 Wild-Type | | $EVI1^{Del190-515}$ | |
|---|---|---|---|---|---|---|---|---|---|---|---|---|---|---|
| | *Fold Change* | *p-value* | *Fold Change* | *p-value* | *Fold Change* | *p-value* | *Fold Change* | *p-value* | *Fold Change* | *p-value* | *Fold Change* | *p-value* | *Fold Change* | *p-value* |
| **Untreated: 0μM HCQ** | 1.0 | - | 1.0 | - | 1.0 | - | 1.0 | - | 1.0 | - | 1.0 | - | 1.0 | - |
| **Untreated: 10μM HCQ** | 0.8 | 0.8818 | 2.5 | 0.0290 | 1.0 | 0.8463 | 4.0 | 0.0009 | 0.6 | 0.0193 | 0.5 | 0.0249 | 0.6 | 0.3266 |
| **Untreated: 25μM HCQ** | 0.8 | 0.8874 | 3.1 | 0.0126 | 0.8 | 0.1027 | 5.9 | 0.0002 | 0.9 | 0.5334 | 0.6 | 0.0413 | 0.7 | 0.4258 |
| **FAC: 0μM HCQ** | 1.7 | 0.2470 | 1.3 | 0.4364 | 0.8 | 0.0782 | 0.8 | 0.5921 | 1.9 | 0.0947 | 2.0 | 0.0482 | 2.5 | 0.0203 |
| **FAC: 10μM HCQ** | 1.5 | 0.4587 | 2.9 | 0.0030 | 0.7 | 0.0129 | 3.5 | 0.0035 | 1.4 | 0.1523 | 1.2 | 0.4304 | 1.4 | 0.3690 |
| **FAC: 25μM HCQ** | 1.8 | 0.2637 | 3.1 | 0.0092 | 0.7 | 0.0133 | 5.8 | 0.0003 | 1.5 | 0.0012 | 1.6 | 0.0066 | 1.8 | 0.1328 |

## D

| Samples | β-catenin | | p62 | | LC3B-I | | LC3B-II | | FANCD2 | | EVI1 Wild-Type | | $EVI1^{Del190-515}$ | |
|---|---|---|---|---|---|---|---|---|---|---|---|---|---|---|
| | *Fold Change* | *p-value* | *Fold Change* | *p-value* | *Fold Change* | *p-value* | *Fold Change* | *p-value* | *Fold Change* | *p-value* | *Fold Change* | *p-value* | *Fold Change* | *p-value* |
| **Untreated** | 1.0 | - | 1.0 | - | 1.0 | - | 1.0 | - | 1.0 | - | 1.0 | - | 1.0 | - |
| **FAC** | 1.5 | 0.1143 | 0.8 | 0.5148 | 0.5 | 0.0748 | 0.7 | 0.1158 | 3.8 | 0.2693 | 2.8 | 0.0693 | 3.1 | 0.0497 |
| **FAC+MG132** | 2.2 | 0.0191 | 1.6 | 0.3307 | 0.9 | 0.5244 | 6.1 | < 0.0001 | 2.3 | 0.5690 | 2.3 | 0.1107 | 2.9 | 0.0690 |
| **FAC+HCQ** | 1.4 | 0.0738 | 1.8 | 0.0881 | 0.5 | 0.0729 | 10.3 | 0.0002 | 2.9 | 0.5796 | 2.0 | 0.0487 | 2.1 | 0.0469 |
| **FAC+HCQ+MG132** | 2.7 | 0.0015 | 1.7 | 0.0915 | 0.6 | 0.0863 | 16.8 | 0.0007 | 2.9 | 0.5872 | 3.4 | 0.0627 | 4.9 | 0.0378 |
